# Analysis of person-hours required for proton beam therapy for pediatric tumors

**DOI:** 10.1093/jrr/rrad022

**Published:** 2023-05-03

**Authors:** Masashi Mizumoto, Hiroko Fukushima, Toshio Miyamoto, Yoshiko Oshiro, Taisuke Sumiya, Takashi Iizumi, Takashi Saito, Hirokazu Makishima, Haruko Numajiri, Sho Hosaka, Kumie Nagatomo, Yuni Yamaki, Kei Nakai, Hideyuki Sakurai

**Affiliations:** Department of Radiation Oncology, University of Tsukuba, 1-1-1 Tennoudai, Tsukuba, Ibaraki 305-8575, Japan; Department of Child Health, Faculty of Medicine, University of Tsukuba, 1-1-1 Tennoudai, Tsukuba, Ibaraki 305-8575, Japan; Department of Radiation Oncology, University of Tsukuba, 1-1-1 Tennoudai, Tsukuba, Ibaraki 305-8575, Japan; Department of Radiation Oncology, University of Tsukuba, 1-1-1 Tennoudai, Tsukuba, Ibaraki 305-8575, Japan; Department of Radiation Oncology, University of Tsukuba, 1-1-1 Tennoudai, Tsukuba, Ibaraki 305-8575, Japan; Department of Radiation Oncology, University of Tsukuba, 1-1-1 Tennoudai, Tsukuba, Ibaraki 305-8575, Japan; Department of Radiation Oncology, University of Tsukuba, 1-1-1 Tennoudai, Tsukuba, Ibaraki 305-8575, Japan; Department of Radiation Oncology, University of Tsukuba, 1-1-1 Tennoudai, Tsukuba, Ibaraki 305-8575, Japan; Department of Radiation Oncology, University of Tsukuba, 1-1-1 Tennoudai, Tsukuba, Ibaraki 305-8575, Japan; Department of Pediatrics, University of Tsukuba, 1-1-1 Tennoudai, Tsukuba, Ibaraki 305-8575, Japan; Department of Pediatrics, University of Tsukuba, 1-1-1 Tennoudai, Tsukuba, Ibaraki 305-8575, Japan; Department of Pediatrics, University of Tsukuba, 1-1-1 Tennoudai, Tsukuba, Ibaraki 305-8575, Japan; Department of Radiation Oncology, University of Tsukuba, 1-1-1 Tennoudai, Tsukuba, Ibaraki 305-8575, Japan; Department of Radiation Oncology, University of Tsukuba, 1-1-1 Tennoudai, Tsukuba, Ibaraki 305-8575, Japan

**Keywords:** pediatrics, proton beam therapy, person-hours, treatment, preparation

## Abstract

Proton beam therapy (PBT) is effective for pediatric tumors, but patients may require sedation and other preparations, which extend the treatment time. Pediatric patients were classified into sedation and non-sedation cases. Adult patients were classified into three groups based on irradiation from two directions without or with respiratory synchronization and patch irradiation. Treatment person-hours were calculated as follows: (time from entering to leaving the treatment room) × (number of required personnel). A detailed analysis showed that the person-hours required for the treatment of pediatric patients are about 1.4–3.5 times greater than those required for adult patients. With the inclusion of additional time for the preparation of pediatric patients, PBT for pediatric cases is two to four times more labor-intensive than for typical adult cases.

## INTRODUCTION

Treatment for pediatric tumors is based on a multidisciplinary approach that combines surgery, chemotherapy and radiation therapy [1]. In recent years, proton beam therapy (PBT) has been recommended for pediatric tumors to reduce late toxicities [2]. However, radiation therapy for pediatric tumors often requires sedation and preparation, and this places a large burden on medical sites [3]. Sedation for patients under 4 years is almost always needed, and therefore, the actual treatment time tends to be longer. In this report, we examined the person-hours required for PBT for adult and pediatric cases to obtain an accurate evaluation of the burden of PBT for pediatric tumors.

## PATIENTS AND METHODS

The subjects were 32 pediatric patients who received PBT at our hospital from January 2010 to April 2011. Data for these patients were compared with those for 90 adult patients who received PBT from January 2022 to November 2022. Written informed consent was obtained in all cases, and the study was approved by the hospital ethics committee (H21–388, Tsukuba Clinical Research & Development Organization). For pediatric cases, the time from entering the irradiation room to leaving the room was measured for each treatment, and the number of staff (radiotherapy technicians and nurses) involved in the treatment was also examined. For adult cases, the number of staff involved in PBT is fixed at three, so only the time from entering to leaving the treatment room was measured. Pediatric patients were classified into sedation and non-sedation cases. Adult patients were classified into three groups based on irradiation from two directions without or with respiratory synchronization, and patch irradiation [4]. Treatment person-hours were calculated as follows: (time from entering to leaving the treatment room) × (number of required personnel).

## RESULTS

Of the 32 pediatric patients in the study, 12 were treated while under sedation. Of the 90 adult patients, 30 patients were treated with and without respiratory synchronization and with patch irradiation. The mean ages of the pediatric patients were 3 (range 1–5) years for those treated with sedation and 5 (3–7) years for those treated without sedation.

The mean treatment times (from entering to leaving the treatment room) were 20.7 min (pediatric cases without sedation), 30.1 min (pediatric cases with sedation), 11.5 min (without respiratory synchronization in adults), 15.6 min (with respiratory synchronization in adults) and 23.7 min (patch irradiation in adults). The mean numbers of personnel were 3.08 and 3.77 for pediatric cases without and with sedation, respectively, and the number of personnel for adult cases was fixed at 3. Based on these results, the treatment person-hours were 64.5 and 120.9 min for pediatric patients without and with sedation, respectively, and 34.3, 46.7 and 71.1 min for adult patients without and with respiratory synchronization, and with patch field irradiation, respectively (Table 1).

**Table 1:** Treatment person-hours according to patient background

Case	*n*	Treatment time (range) (min)^a^	Required personnel (number)	Person-hours (per treatment day) (min)	Ratio^b^
Pediatric, non-sedation	20	20.7 (8–77)	3.08	64.5	1.9
Pediatric, sedation	12	30.1 (10–145)	3.77	120.9	3.5
Adult, non-respiratory synch	30	11.5 (9–14)	3.00	34.3	1
Adult, respiratory synch	30	15.6 (11–28)	3.00	46.7	1.4
Adult, patch irradiation	30	23.7 (12–34)	3.00	71.1	2.1

^a^Treatment time measured from entering to leaving the treatment room.

^b^Relative to adult cases without respiratory synchronization.

In pediatric patients, preparation procedures were performed five times on average (range 0–25) before the start of and during PBT. For sedated cases compared with non-sedated cases, the heart rate and oxygen saturation need to be monitored during the irradiation, and the final adjustments of sedation are also required after the patient has been moved to the treatment bed. It usually takes an additional 5–10 min to perform both steps.

The preparation person-hours calculated as [(all preparation time) × (required personnel)]/(irradiation time) were 14.3 min for cases without sedation and 20.5 for those with sedation. The total person-hours, including the time for preparation, are shown in Table 2. In the clinical setting, it is also necessary for a pediatrician and a nurse to accompany a sedated patient during movement from the pediatric ward to the PBT facility, but this is not included in the analysis.

**Table 2:** Total person-hours according to patient background

Case	*n*	Treatment person-hours (min)^a^	Preparation person-hours (min)	Total person-hours (min)	Ratio^b^
Pediatric, non-sedation	20	64.5	14.3	78.8	2.3
Pediatric, sedation	12	120.9	20.5	141.4	4.1
Adult, non-respiratory synch	30	34.3	0	34.3	1
Adult, respiratory synch	30	46.7	0	46.7	1.4
Adult, patch irradiation	30	71.1	0	71.1	2.1

^a^Treatment time measured from entering to leaving the treatment room.

^b^Relative to adult cases without respiratory synchronization.

## DISCUSSION

PBT has an excellent dose concentration due to its focused energy peak and, therefore, is widely used for various tumors [5–9]. For pediatric cases and adolescent and young adult patients, PBT is favored over photon radiotherapy due to reduced future adverse effects and secondary cancer [2,10–14]. However, treatment for pediatric patients requires more time and effort for sedation and alignment compared with that for adult patients, which is a major negative point in terms of economic efficiency.

In this study, the treatment person-hours for PBT for pediatric cases without sedation were 1.9, 1.4 and 0.9 times greater than those for adult patients irradiated without and with breathing synchronization, and with patch irradiation, respectively, and 3.5, 2.6 and 1.7 times greater for pediatric cases with sedation compared with the respective adult cases. Given that patch irradiation is a rare and non-standard method, the treatment person-hours for PBT for a pediatric tumor are generally 1.4–3.5 times greater than those for adult patients. We note that the study period differed between pediatric (2010–11) and adult (2022) cases, but the treatment equipment, irradiation technique and number of medical staff were similar.

Various preparation procedures are used for irradiation with PBT for pediatric tumors [15–18]. With the inclusion of preparation person-hours, treatment of a pediatric case without sedation took about twice as much time as treatment of adult patients (total person-hours: 78.8 vs 34.4–46.7 min) and treatment of a pediatric case with sedation took three to four times more than that for adult patients (141.4 vs 34.3–46.7 min). In addition, one pediatrician and one nurse needed to make a round trip from the ward to the treatment room for sedated patients (about 20–30 min per irradiation). The time required for this activity was not considered in the analysis; therefore, more person-hours are actually used for pediatric patients who need sedation. Given these transportation person-hours, PBT for pediatric cases in clinical practice is at least two to four times more labor-intensive than that for typical adult cases. However, this treatment is particularly effective in these cases, and further implementation of PBT requires increased support from facilities.

In conclusion, the results of this study indicate that PBT for pediatric cases is much more labor-intensive than for adult cases.

## CONFLICT OF INTEREST

None declared.

## FUNDING

This work was supported by the University of Tsukuba.

## DATA AVAILABILITY

Research data are stored in an institutional repository and will be shared upon request to the corresponding author.
